# Circulating miRNAs as Promising Biomarkers to Evaluate ECMO Treatment Responses in ARDS Patients

**DOI:** 10.3390/membranes11080551

**Published:** 2021-07-22

**Authors:** Gennaro Martucci, Antonio Arcadipane, Fabio Tuzzolino, Giovanna Occhipinti, Giovanna Panarello, Claudia Carcione, Alessandro Bertani, Pier Giulio Conaldi, Vitale Miceli

**Affiliations:** 1 Anesthesia and Intensive Care Department, IRCCS-ISMETT, 90127 Palermo, Italy; gmartucci@ismett.edu (G.M.); aarcadipane@ismett.edu (A.A.); gocchipinti@ismett.edu (G.O.); gpanarello@ismett.edu (G.P.); 2 Research Department, IRCCS-ISMETT, 90127 Palermo, Italy; ftuzzolino@ismett.edu (F.T.); pgconaldi@ismett.edu (P.G.C.); 3 Fondazione Ri.MED, 90127 Palermo, Italy; ccarcione@fondazionerimed.com; 4 Division of Thoracic Surgery and Lung Transplantation, Department for the Treatment and Study of Cardiothoracic Diseases and Cardiothoracic Transplantation, IRCCS-ISMETT, 90127 Palermo, Italy; abertani@ismett.edu

**Keywords:** ARDS, ECMO, biomarkers, miRNAs

## Abstract

The use of extracorporeal membrane oxygenation (ECMO) for acute respiratory distress syndrome (ARDS) has increased in the last decade. However, mortality remains high, and the complexity of ECMO requires individualized treatment. There are some biomarkers to monitor progression and predict clinical outcomes of ARDS. This project aims to advance the management of ARDS patients treated with ECMO by exploring miRNA expression in whole blood. The analysis was conducted on two groups with different length of ECMO: Group A (longer runs) and group B (shorter runs). We analyzed miRNAs before ECMO cannulation, and at 7 and 14 days of ECMO support. Our results showed that in the group B patients, 11 deregulated miRNAs were identified, and showed an opposite trend of expression compared to the group A patients. In silico analysis revealed that these 11 miRNAs were related to processes involved in the pathogenesis and evolution of ARDS. This scenario could represent homeostatic mechanisms by which, in ECMO responsive patients, pathways activated during ARDS progression are switched-off. Circulating miRNAs could represent promising biomarkers to monitor the evolution of ARDS under ECMO support. Further studies may shed light on this topic to improve a personalized approach in such a complex setting of patients.

## 1. Introduction

The use of veno-venous extracorporeal membrane oxygenation (ECMO) is an established tool in the most severe cases of patients with acute respiratory distress syndrome (ARDS). ECMO provides a temporary circulatory support that can substitute the lung function for days to months if mechanical ventilation (MV) cannot adequately guarantee gas exchanges or the risk of ventilator-induced lung injury is too high. In a wide clinical view, ECMO aims at allowing the “lung rest” by lowering airway pressures and tidal volumes. However, despite numerous advances in ECMO treatment, the mortality rate of patients remains unacceptably high [1,2].

The wide use of ECMO is derived both from the improvements of ECMO technology and the increased knowledge acquired since the 2009 H1N1 pandemic [3,4]. Despite a recent randomized clinical trial [5], its efficacy and benefits are still questioned. Indeed, the mortality rates are very variable [2,6,7,8], and it has been highlighted that an appropriate patient selection and experience in ECMO management are the keys for successful treatment [9].

Though several studies have shown that many scoring systems used in the intensive care unit (ICU) can predict the outcomes for ECMO patients [10,11], those scorings need a number of laboratory data integrated with physiological parameters, are sometimes too complex to calculate, and have a low grade of predictivity. On the other hand, several biomarkers have been applied to determine neurological and renal dysfunction as outcome predictors of ECMO treatment [12,13]. Moreover, it has been shown that serum biomarkers of endothelial injury and activation might represent prognostic factors in this setting [14].

MicroRNAs (miRNAs) are non-coding RNA with gene/pathway-regulatory function [15], and their altered expression in biological fluids such as blood has been linked to the presence of many disease states, including ARDS, in which a diffuse alveolar damage is determined by the activation of inflammation and apoptosis pathways [16]. Moreover, during ECMO, several coagulative and inflammatory cascades similar to systemic inflammatory response syndrome (SIRS) are activated when the patient’s blood is exposed to the surface of the ECMO circuit [17]. Different miRNAs are able to regulate both platelet activation and crucial proteins involved in the regulation of hemostatic processes [18,19]. miRNA biomolecules also seem to play an important role in the regulation of gene expression during the pathogenesis of ARDS, being deregulated in pulmonary and extrapulmonary ARDS patients [20], as well as in patients with different severity status of ARDS [21].

To date, there are some available biomarkers to monitor progression and to predict outcomes of ECMO patients. On the other hand, there is a lack of standardized markers to measure the evolution of ARDS and detect the nuances among the patients of a very severe population of the critically ill. In this paper, to obtain future reliable markers, we preliminarily tested the hypothesis that whole blood miRNAs are differently regulated during ECMO according to a shorter or longer ECMO stay.

## 2. Materials and Methods

### 2.1. Characteristics of Patients and Sample Acquisition

This study was approved by the IRCCS-ISMETT Ethics Committee (EC Code: IRRB/34/19) and conducted in accordance with the ethical standards laid down in the 1964 Declaration of Helsinki and its later amendments. Written informed consent was initially obtained from the next of kin and later confirmed directly by the patients, if recovered. We enrolled ARDS patients supported by ECMO and performed miRNA measurement for this study. Patients were recorded for anthropometric data: age, weight, height, and body mass index (BMI), severity of disease (Simplified Acute Physiology Score (SAPS II)), Sequential Organ Failure Assessment (SOFA) score, creatinine, hematocrit (HTC), bilirubin, PaO_2_/FiO_2_ ratio, and ECMO related score. Moreover, the length of ECMO course was evaluated and used to stratify patients into two groups: group A (late ECMO responders) and group B (early ECMO responders).

The weaning from ECMO does not currently follow a standardized protocol, but some principles, also highlighted in recent literature, should be verified before decannulation: (1) patient has to be recovered or relevantly improved at chest x ray; (2) mechanical ventilation should be eventually still needed but with a protective strategy still in place—preferably 6–8 mL/kg of ideal body weight, driving pressure ≤ 15 cm H_2_O, and respiratory frequency less than 25 breaths per minute; (3) hemodynamics should be stable with a heart rate less than 110 beats per minute; (4) gas exchanges should be in normal range with progressive reduction of need for blood flow and sweep gas flow to be maintained; (5) a cycle of complete ECMO off (sweep gas flow off as well as any FiO_2_) should be tolerated for at least 24 h for ECMO lasting 7–10 days, and a complete ECMO off tolerated for 48 h in the case of longer ECMO stay [22,23,24].

For miRNA evaluation, whole blood was collected in PAXgene tubes (Qiagen, Hilden, Germany) before cannulation (pre-ECMO), and after 7 and 14 days of ECMO. Unprocessed samples were immediately stored at −80 °C until further analysis.

### 2.2. miRNA Expression Profiling

We analyzed miRNA expression with TaqMan^®^ low-density arrays (TLDA) (TaqMan^®^ Array Human MicroRNA A+B Cards Set v3.0) according to the manufacturer’s instructions (Thermo Fisher Scientific, Waltham, MA, USA). Total RNA was extracted with RiboPure™ RNA Purification Kit, blood (ThermoFisher Scientific, Waltham, MA, USA). The purity and quantity of isolated RNA were determined by OD260/280 using a NanoDrop Spectrophotometer (Thermo Fisher Scientific, Waltham, MA, USA). The RNA integrity of all samples was assessed by calculating RIN values using the 4200 TapeStation System (Agilent technologies, Santa Clara, CA, USA). Then, 300 ng of RNA was reverse-transcribed with the high-capacity RNA-to-cDNA kit according to the manufacturer’s instructions (Thermo Fisher Scientific, Waltham, MA, USA). We analyzed the expression of 754 human miRNAs with the Applied Biosystems 7900 Real-Time PCR (Thermo Fisher Scientific, Waltham, MA, USA). The fold change in miRNA expression was determined according to the 2^−ΔΔCt^ method, using the U6 as reference gene. Hierarchical cluster analysis of miRNA expression was used to group patients at the same treatment time. miRNA expression data were grouped using the Cluster3.0 program, and a heat map was generated using the Java TreeView program.

### 2.3. miRNAs Functional Analysis

We performed miRNA enrichment analysis on differentially expressed miRNAs with DIANA miRpath v3.0 [25] (http://snf-515788.vm.okeanos.grnet.gr/, date of access: 31 March 2021), and the miRNet online database [26] (https://www.minet.ca/faces/home.xhtml, date of access: 31 March 2021). Using known or predicted interactions between miRNAs and target genes, each software allowed for the elucidation of molecular pathways controlled by miRNAs. In particular, with DIANA miRPath, our investigation was based on the microT-CDS algorithm; we performed Gene Ontology (GO) analysis and merged results by miRNAs and pathways with default settings. The miRNet database is a comprehensive atlas of miRNA–target interactions that can integrate the information resulting from 11 existing miRNA–target prediction programs (TarBase, miRTarBase, miRecords, miRanda, miR2Disease, HMDD, PhenomiR, SM2miR, PharmacomiR, EpimiR, and starBase). The software uses standard enrichment analysis based on the hypergeometric tests after adjustment for false discovery rate (FDR). With miRNET we investigated the functional implications of miRNA deregulation in ECMO-treated ARDS patients and generated a protein–protein interaction network of proteins targeted by deregulated miRNAs and involved in crucial pathways of ARDS pathogenesis.

### 2.4. Statistical Analysis

Anthropometric and clinical characteristics of patients are reported as medians and 25th and 75th percentiles for continuous data and frequencies, and percentages for categorical data. To test the significant differences of miRNA expression between samples, we performed multi-step statistical tests with both a parametric Student’s t-test and a non-parametric Mann–Whitney U-test. An appropriate paired or unpaired Student’s t-test was used for the comparison of fold change values (2^−ΔΔCt^). We also analyzed multiple pair-wise comparisons of each 2^−ΔCt^ value per group through both one-way ANOVA with a post hoc Tukey test and Mann–Whitney U test (GraphPad Prism 6.0, San Diego, CA USA). *p* ≤ 0.05 was considered as significant. We clustered and correlated data using hierarchical clustering and Euclidean distance algorithms. Both sensitivity and specificity of miRNAs to discriminate between the two groups of patients were assessed with receiver operating characteristic (ROC) curve analysis.

## 3. Results

### 3.1. Patients Characteristics and Stratification

We collected blood samples from ten consecutive ARDS patients supported by ECMO. Causes of ARDS were viral pneumonia (n = 3), bacterial pneumonia (n = 6), and severe polytrauma with pulmonary contusion and bacteria lover-infection (n = 1). Baseline anthropometric and clinical characteristics of the overall population are shown in Table 1. All the patients were screened before cannulation by transthoracic echocardiogram and no relevant abnormalities were found with an ejection fraction of at least 50% in all the patients. In order to identify deregulated miRNAs related to the efficacy of ECMO treatment, considering the usual duration of the treatment (7–10 days) in patients with ARDS who benefit from it [27], we stratified patients into two groups based on ECMO duration: group A, with longer ECMO support (median 40 days) and group B, with short ECMO support (median 9 days). Patient characteristics at the baseline showed no statistically significant differences between the two groups (all *p*-values > 0.05), showing a homogeneous cohort at least from the clinical standpoint (Table 1). Moreover, during the ECMO stay no relevant complications related to ECMO were recognized, and the clinical reason for longer ECMO stay was principally the lack or slow recover of pulmonary function.

### 3.2. Differentially Expressed miRNAs in Patients with Long and Short ECMO Duration

**Figure 1:**
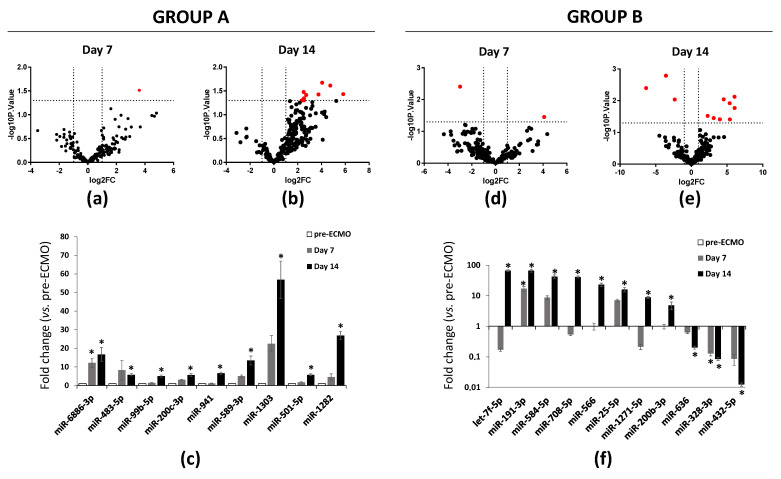
RT-PCR analysis of miRNAs in ARDS patients supported with ECMO for 14 days. (**a**) Volcano plot analysis (*p* ≤ 0.05) plot analysis of RNAs in ARDS patients supported with ECMO group A patients after 7 and (**b**) 14 days of ECMO support. (**c**) Up-regulated miRNAs in group A patients after 7 and 14 days of ECMO support. (**d**) Volcano plot analysis (*p* ≤ 0.05 and fold change ≥ 0) of deregulated miRNAs analyzed in group B patients after 7 and (**e**) 14 days of ECMO support. (**f**) Up- and down-regulated miRNAs in group B patients after 7 and 14 days of ECMO support. miRNA levels were normalized to those of U6. Data are means ± SD. (Comparisons made by Student’s *t*-test). * *p* < 0.05 vs. pre-ECMO (patients before cannulation).

To identify differentially expressed miRNAs during ECMO, we analyzed their expression in group A and B at the study time points. Analyses were carried out on 229 miRNAs selected from 754 miRNAs after quality control screening (amplification score > 1.1, Cq confidence > 0.8). After differential analysis with Student’s t-test, in group A, by volcano plot analysis (*p* < 0.05 and fold change > 2), we identified one up-regulated miRNA and nine up-regulated miRNAs after 7 and 14 days of ECMO treatment, respectively (Figure 1a–c). In group B, one miRNA was up-regulated, and one miRNA was down-regulated after 7 days of ECMO treatment, while eight miRNAs were up-regulated and three miRNAs were down-regulated after 14 days (Figure 1d–f). The subsequent analyses were directed on those.

**Figure 2:**
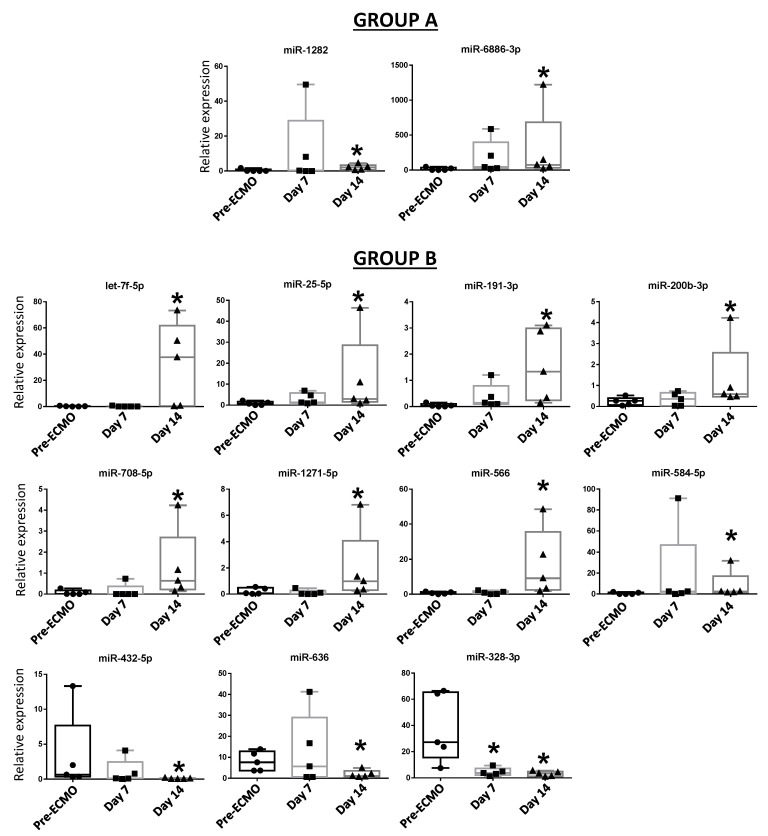
Blood expression levels of thirteen miRNAs in group A and group B patients after 7 and 14 days of ECMO support. Data are presented as expression relative to U6. Box plots are displayed where the horizontal bar represents the median, the box represents the IQR, and the whiskers represent the maximum and minimum values. Comparisons were made by Mann–Whitney U test. * *p* < 0.05.

We then conducted differential analysis with the Mann–Whitney U test and identified two miRNAs as significantly differentially expressed in group A patients, while 11 deregulated miRNAs were identified in group B (Figure 2). In particular, miR-1282 and miR-6886 were significantly up-regulated after 14 days of ECMO support in group A. In group B, let-7f, miR-25, miR-191, miR-200b, miR-708, miR-1271, miR-566, and miR-584 were significantly up-regulated after 14 days of ECMO support, while miR-432 and miR-636 were significantly down-regulated after 14 days, and miR-328 was significantly down-regulated after both 7 and 14 days (Figure 2).

### 3.3. Enrichment of the Deregulated miRNAs in Biological Processes

In order to find associations between the aforementioned miRNAs and the evolution of ARDS, we conducted a GO enrichment analysis with the DIANA database. No relevant pathways were found with the examination of the two deregulated miRNAs in group A. On the other hand, the in silico analysis of the 11 deregulated miRNAs of patients in group B revealed that, in the biological process category, most of the miRNAs were related to crucial processes potentially involved in the pathogenesis of ARDS.

**Figure 3:**
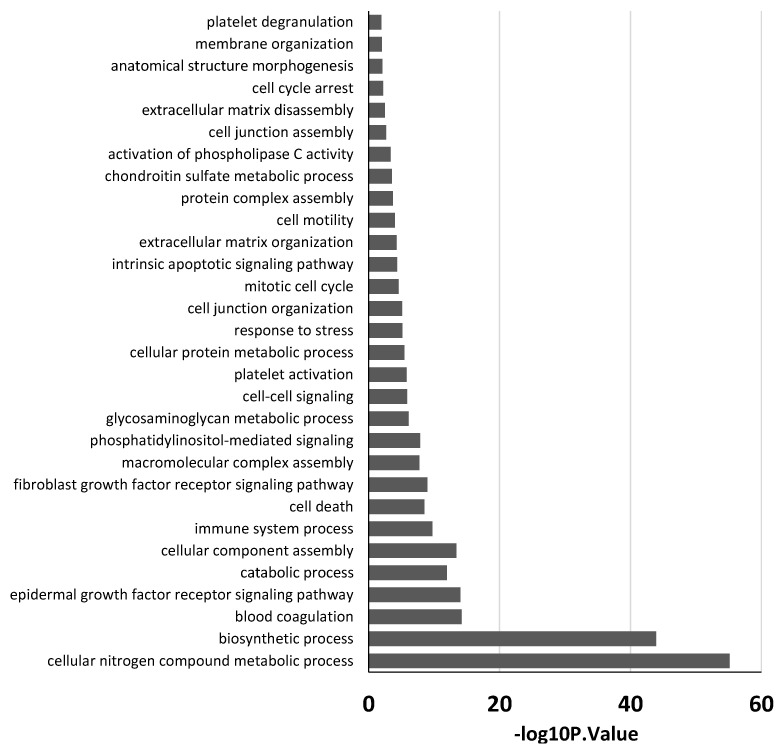
GO analysis of eleven miRNAs deregulated in group B patients. Partial list of biological process enrichment analysis.

In particular, different terms are involved in the regulation of tissue remodeling. Moreover, specific terms were involved in the regulation of both coagulation and immune system (Figure 3).

### 3.4. miRNA Target Network Construction and Analysis of ARDS-Related miRNAs in Group B Patients

**Figure 4:**
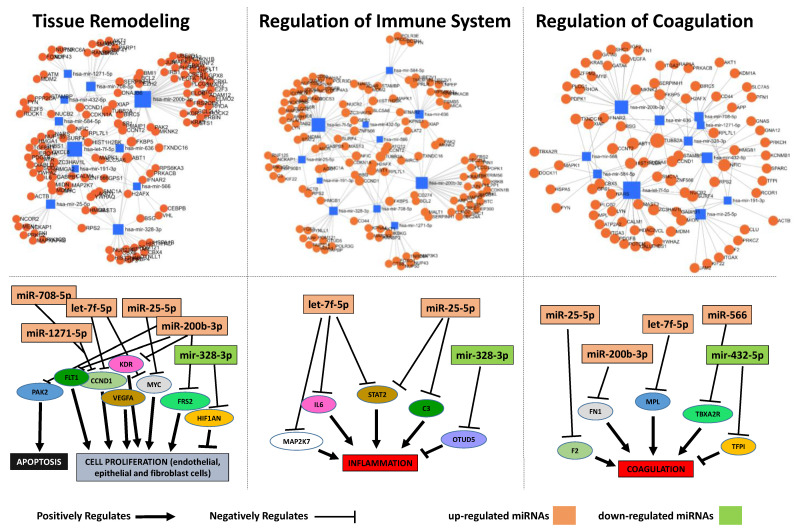
Protein–protein interaction network generated for shared miRNA target genes after miRNET analysis. Top images show networks with all interactions between deregulated miRNAs and genes involved in tissue remodeling, regulation of the immune system and regulation of coagulation. Bottom images show schematic representation of crucial proteins involved in the regulation of each pathway and their interaction with some deregulated miRNAs.

**Figure 5:**
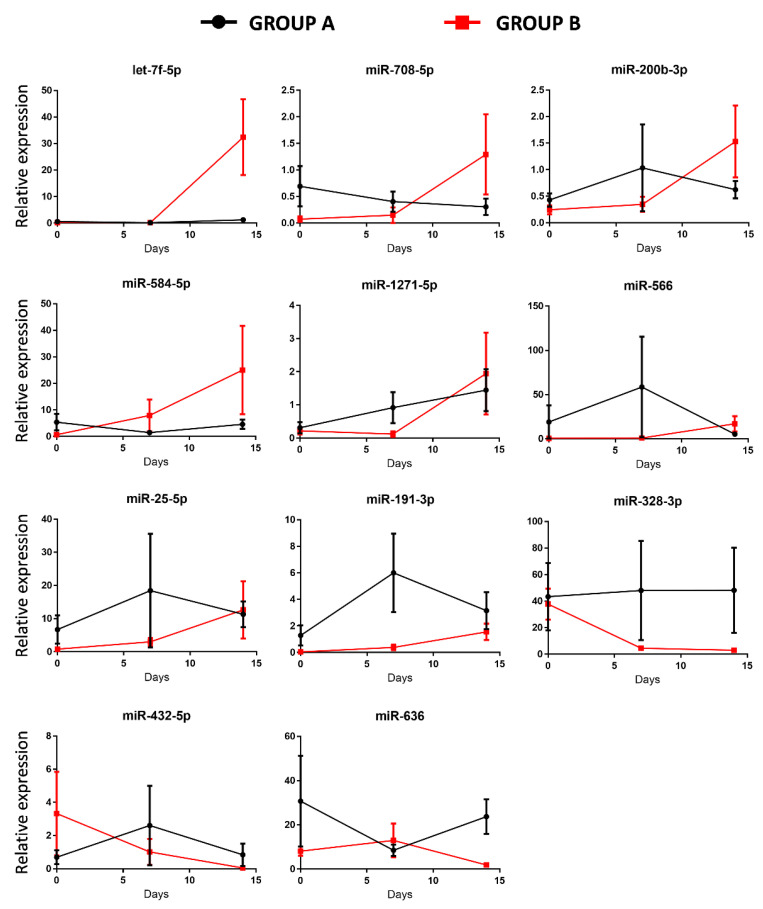
Temporal expression of miRNAs after 7 and 14 days of ECMO support in group A and in group B patients. Data shown are mean values ± SE of miRNA expression of 10 patients for each time point.

To explore whether the 11 differentially expressed miRNAs of patients in group B can be involved in the regulation of the ARDS-related pathways, we also analyzed them using the miRNET database. A miRNA–target network of representative pathways involved in the pathogenesis of ARDS was analyzed: 1. Tissue remodeling; 2. Regulation of immune system; 3. Regulation of coagulation. The selected 11 miRNA (let-7f, miR-25, miR-191, miR-200b, miR-708, miR-1271, miR-566, miR-584, miR-432, miR-636, and miR-328) target genes were implicated in all aforementioned pathways (Figure 4 top and Table 2). Interestingly, some miRNAs, such as let-7f, miR-708, miR-200b, miR-584, miR-328, and miR-636, showed an evident opposed trend of expression after 14 days of ECMO support between group A and B (Figure 5).

**Figure 6:**
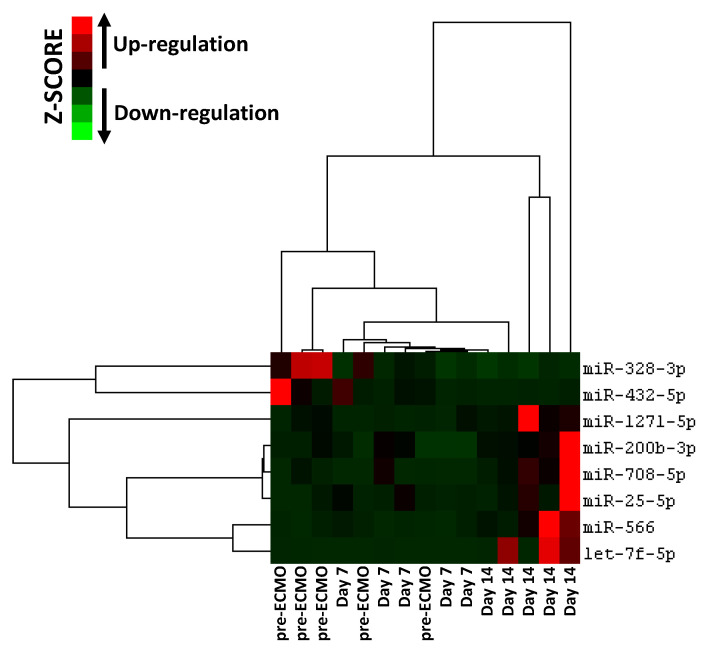
Hierarchical clustering analysis based on eight selected miRNA expression levels in ARDS patients supported with ECMO for 14 days. Heatmap colors represent relative miRNA expression normalized to housekeeping.

Furthermore, we carried out hierarchical cluster analysis of the eight miRNAs that target crucial proteins involved in the regulation of major pathways implicated in ARDS (Figure 4 bottom). The analysis revealed that those miRNAs were able to predict ARDS progression in relation to their expression (Figure 6).

**Figure 7:**
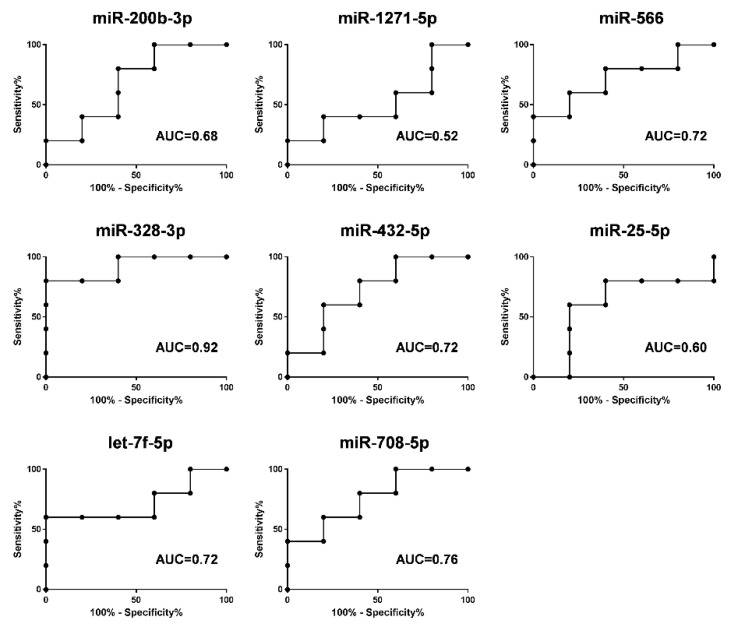
ROC curves of the diagnostic potential of the individual blood miRNAs (miR-200b-3p, miR-1271-5p, miR-566, miR-328-3p, miR-432-5p, miR-25-5p, let-7f-5p and miR-708-5p) after 14 days of ECMO support discriminating between group A and group B patients. ROC curves were made with values of relative expression of each miRNA at 14 days of ECMO support. The area under curve (AUC) values ranged from 0.52 to 0.92.

To evaluate the diagnostic power of the eight selected miRNAs, we performed ROC curve analyses for detection of ECMO efficacy. A scoring approach revealed a good diagnostic accuracy with an AUC ranged from 0.52 to 0.92 (Figure 7).

## 4. Discussions

In this study, we preliminarily tested the hypothesis that circulating miRNAs might help to identify the proper ECMO management for different ARDS patients. Therefore, we recognized a different regulation for 11 miRNAs between patients with a short ECMO stay and patients with a longer ECMO duration.

Because of the extensive use of ECMO treatment, there is a need to allocate appropriate ECMO support for each patient. Indeed, despite impressive developments in the field, significant complications, such as thrombosis and bleeding, commonly occur despite anticoagulation [28,29]. All this strongly suggests the need for a personalized approach. miRNAs are altered in both physiological and pathological conditions [20,30,31,32,33], and circulating miRNAs are stable molecules found in blood that represent promising biomarkers in many disorders, including lung diseases [21,34]. In fact, miRNAs are candidate biomarkers to monitor clinical conditions that may require ECMO support [35,36]. They might also be considered indicators of disease progression/resolution.

This study is aimed at exploring whether deregulated miRNAs can be indicative of disease progression in two groups of patients that differ for a different response to ECMO support: group A (late ECMO responders with a median of 40 days) and group B (early ECMO responders with a median of 9 days). Using high-throughput qPCR, based on previous works demonstrating an association between a specific miRNA profile and ARDS [16,20,21,37,38], we screened 754 circulating miRNAs extracted from the whole blood of 10 patients before ECMO cannulation, and 7 and 14 days after ECMO support. To the best of our knowledge, only one study has investigated the potential role of serum circulating miRNAs as biomarkers to predict hematological complications and outcomes in ECMO treated patients. In that study, it was shown that the expression of miRNAs was not altered by ECMO support itself [39]. In our study, we used whole blood samples to explore both circulating exosomal and non-exosomal blood miRNAs [40]. Furthermore, considering that the usual ECMO duration in ARDS patients is 7–10 days [27], differently from Scettri and colleagues, who examined the expression of miRNAs after both 24 and 72 h [39], we analyzed miRNA expression after 7 and 14 days of ECMO support. We found that ECMO itself did not influence the expression of circulating miRNAs in patients with long ECMO runs, while 11 miRNAs were significantly deregulated in shorter ECMO runs. In particular, our retrospective analysis showed no significant deregulated miRNAs after 7 days of ECMO treatment while, mainly in a group of patients with a low median of ECMO duration (group B), 11 miRNAs (let-7f, miR-25, miR-191, miR-200b, miR-708, miR-1271, miR-566, miR-584, miR-432, miR-636, and miR-328) were deregulated after 14 days (Figure 2). GO enrichment analysis revealed that those miRNAs were related to ARDS pathways affected during ECMO treatment (Figure 3). Instead, in another group of patients with a median ECMO duration of 40 days (group A), only two miRNAs (miR-1282 and miR-6886) were significantly deregulated after 14 days, and the GO enrichment analysis for those miRNAs revealed no relation to ARDS pathways.

We hypothesized that ECMO treatment in group B patients affected the evolution of ARDS-related pathways. Therefore, we performed an in silico analysis of the 11 deregulated miRNAs found in group B, and observed that those miRNAs were related to major pathways involved in ARDS: (1) Tissue remodeling; (2) Regulation of immune system; (3) Regulation of coagulation (Figure 4 top). The analysis also revealed that crucial proteins regulating the aforementioned pathways are targeted by eight deregulated miRNAs (Figure 4 bottom). During ECMO, due to both ARDS pathogenesis [17,41] and the continuous contact surface between the blood and the ECMO circuit [18], different coagulative pathways were also regulated by F2, FN1, MPL, and TBXA2R protein (which are known as positive regulators of these pathways) [18,42,43,44] and tissue factor pathway inhibitor (TFPI) (a negative regulator of coagulation processes) [45]. We found that let-7f, miR-25, miR-200b and miR-566 were up-regulated after 14 days of ECMO treatment in group B patients, and those miRNAs target the aforementioned positive regulators. Conversely, TFPI, a negative regulator of coagulation, was targeted by miR-432, which appears to be down-regulated after 14 days of ECMO treatment, (Figure 4 bottom). Moreover, crucial proteins involved in the regulation of the immune system seem to be controlled by our deregulated miRNAs. Indeed, MAP2K7, IL6, STAT2, and C3 protein, which positively regulate immune responses [46,47,48], were targeted by up-regulated miRNAs such as let-7f and miR-25, while the OTUD5 protein (negative regulator of inflammation) [49] was targeted by down-regulated miRNAs, such as miR-328. It has been shown that pulmonary alveolar tissue is subject to remodeling during the evolution of ARDS. In this context, PAK2, FLT1, CCND1, VEGFA, KDR, MYC, and FRS2 have been found to be involved in the two major pathways implicated in tissue remodeling, such as apoptosis and cell proliferation [50,51,52,53,54,55]. We found that those proteins were targeted by let-7f, miR-25, miR-200b, miR-708, and miR-1271, which we observed being up-regulated after 14 days of ECMO support, while HIF1AN, a negative regulator of proliferation [56], is targeted by miR-328, which is down-regulated.

Therefore, the deregulation of eight circulating miRNAs during ECMO support in the group B patients can regulate inflammation, coagulation, and tissue remodeling that govern tissue injury and the repair process of lung parenchyma in ARDS patients. Furthermore, as shown in Figure 6, those miRNAs can help classify ARDS patients in relation to the timing of their ECMO course, and we were able to reach a discriminatory power with AUC ranged from 0.52 to 0.92 (Figure 7). Our results are also in line with several studies that show that some of our deregulated miRNAs were implicated in ARDS pathogenesis [16,30,57]. In addition, the present data sustain, at the miRNA level (at a deeper cell and system interaction), the clinical evidence that during ARDS and during ECMO, a consistent activation of pro-coagulant and anticoagulant pathways coexist at the same time. The clinical meaning of these observations is still to be grasped but, potentially, knowledge on the topic may contribute to understanding, in the single patient, a different behavior unmasked by standard laboratory data.

This is a pilot study intended to serve as the basis for a larger investigation designed for an accurate profile of miRNAs as biomarkers for ECMO monitoring in ARDS patients. We acknowledge several limitations in our study. First, the sample is quite small, and despite a complete similarity between the two studied groups this may have altered the results. The female gender is poorly represented and, therefore, the miRNAs may be biased by the high prevalence of males. Second, the consecutive patients all survived; consequently, we may have chosen the length of stay in ECMO as a parameter strongly different from overall survival on ECMO, despite the fact that duration of ECMO seems to have a clinical significance. Third, we analyzed blood samples 14 days after cannulation, but some patients in the group B were supported with ECMO for less than 14 days, which may have altered the results.

The assessment of patients during ECMO support is imperative in order to select the most appropriate therapeutic approach, which may lead to improvement in the quality and timing of treatment itself. Though ECMO technology has improved survival, many fields of its management are still unknown. Thus, miRNAs can serve as biomarkers for the monitoring of ECMO course, and the identification of patient responses to ECMO can contribute to increasing the precision of ARDS patient management.

## Figures and Tables

**Table 1:** Patients’ anthropometric and clinical characteristics according to ECMO duration outcome.

Variable	Overall	Group A (n = 5)	Group B (n = 5)	*p*-Value
Gender, N (%)	Male 8 (80%) Female 2 (20%)	Male, 4 (80%) Female, 1 (20%)	Male, 4 (80%) Female, 1 (20%)	>0.9
Age (years)	50.7 (±15.04)	44.6 (±18.57)	56.8 (±8.44)	0.217
Causes of ARDS (%)				
Viral pneumonia	3 (30%)	2 (20%)	1 (10%)	0.698
Bacterial pneumonia	6 (60%)	2 (20%)	4 (40%)	0.698
Polytrauma with bacterialover-infection	1 (10%)	1 (10%)	0 (0%)	0.423
BMI (kg/m^2^)	28.3 (26.2, 28.7)	29 (26.6, 34.8)	26.5 (26.2, 27.7)	0.174
SAPS II (Admission)	37.5 (34.5, 54.7)	34 (32, 45)	39 (36, 58)	0.548
SOFA (Admission)	5.5 (3.2, 9.5)	6 (4, 10)	5 (3, 8)	0.603
RESP Score (Admission)	0.5 (−2.5, 4.7)	4 (−1, 5)	−1 (−3, 2)	0.572
PaO2/FiO2 PRE-ECMO (mmHg)	60.5 (56.2, 68.5)	61 (60, 70)	60 (55, 64)	0.573
Creatinine (mg/dL)	1.48 (0.85, 3.17)	0.8 (0.6, 1.6)	2.8 (1.36, 3.3)	0.291
HTC (%)	30.3 (30, 38.3)	30.5 (30.2, 40)	30 (30, 33.2)	0.972
Bilirubin (mg/dL)	0.96 (0.79, 1.28)	0.86 (0.77, 1.3)	1 (0.92, 1.23)	0.811
ECMO Duration (Days)	25.5 (11.5, 39.7)	40 (39, 65)	9 (9, 19)	0.022

Continuous variables are presented as median value (25th to 75th percentile range) or mean ± SD, and nominal variables are presented as absolute quantity (percentage). BMI: body mass index; SAPS II Score: Simplified Acute Physiology 2 score; SOFA score: Sequential Organ Failure Assessment score; RESP score: Respiratory Extracorporeal Membrane Oxygenation Survival Prediction score; PaO_2_:FiO_2_: ratio of fraction of partial pressure of O_2_ to inspired O_2_; HTC: Hematocrit; ECMO: Extracorporeal membrane oxygenation.

**Table 2:** Top ranked miRNAs and genes found in the network analysis.

Tissue Remodeling		Regulation of Immune System		Regulation of Coagulation	
Top miRNAs	Genes	Top miRNAs	Genes	Top miRNAs	Genes
hsa-mir-200b-3p	47	hsa-let-7f-5p	44	hsa-let-7f-5p	30
hsa-let-7f-5p	37	hsa-mir-200b-3p	36	hsa-mir-200b-3p	20
hsa-mir-328-3p	20	hsa-mir-636	19	hsa-mir-25-5p	13
hsa-mir-708-5p	16	hsa-mir-328-3p	18	hsa-mir-432-5p	12
hsa-mir-25-5p	15	hsa-mir-25-5p	14	hsa-mir-636	11
hsa-mir-636	11	hsa-mir-708-5p	14	hsa-mir-328-3p	9
hsa-mir-584-5p	10	hsa-mir-584-5p	13	hsa-mir-566	9
hsa-mir-432-5p	8	hsa-mir-1271-5p	11	hsa-mir-708-5p	9
hsa-mir-566	8	hsa-mir-566	8	hsa-mir-584-5p	8
hsa-mir-1271-5p	8	hsa-mir-432-5p	7	hsa-mir-191-3p	6
hsa-mir-191-3p	6	hsa-mir-191-3p	6	hsa-mir-1271-5p	5

## Data Availability

The datasets used and analyzed are available from the corresponding author on reasonable request.
